# Draft Genome Sequence of *Saccharomonospora piscinae* KCTC 19743^T^, an Actinobacterium Containing Secondary Metabolite Biosynthetic Gene Clusters

**DOI:** 10.1128/MRA.01588-19

**Published:** 2020-04-09

**Authors:** Ninfa Ramírez-Durán, Rafael R. de la Haba, Blanca Vera-Gargallo, Cristina Sánchez-Porro, Scarlett Alonso-Carmona, Horacio Sandoval-Trujillo, Antonio Ventosa

**Affiliations:** a Faculty of Medicine, Autonomous University of the State of Mexico, Toluca, Mexico; b Department of Microbiology and Parasitology, Faculty of Pharmacy, University of Seville, Seville, Spain; c Department of Biological Systems, Metropolitan Autonomous University-Xochimilco, Mexico City, Mexico; University of Southern California

## Abstract

The draft genome sequence of *Saccharomonospora piscinae* KCTC 19743^T^, with a size of 4,897,614 bp, was assembled into 11 scaffolds containing 4,561 open reading frames and a G+C content of 71.0 mol%. Polyketide synthase and nonribosomal peptide synthetase gene clusters, which are responsible for the biosynthesis of several biomolecules, were identified and located in different regions in the genome.

## ANNOUNCEMENT

The actinobacterial group has been recognized for its extensive secondary metabolism, and members produce approximately two-thirds of all antibiotics used in clinical, industrial, and biotechnological processes (1). There are two classes of bacterial bioactive secondary metabolites, namely, the polyketides and the nonribosomal peptides, which are biosynthesized by multifunctional enzymes, i.e., polyketide synthases (PKSs) and nonribosomal peptide synthetases (NRPSs), respectively (2).

Saccharomonospora piscinae KCTC 19743^T^ is the type strain of the most recently described species of the genus *Saccharomonospora*. It was isolated from the sediment of a fishpond in southern Taiwan, and it is characterized by its ability to grow at 0 to 8% (wt/vol) NaCl and between 20°C and 40°C (3). The aims of this work were to obtain the genome sequence of Saccharomonospora piscinae KCTC 19743^T^ and to determine the presence of secondary metabolite biosynthetic gene clusters.

The type strain of Saccharomonospora piscinae was obtained from the Korean Collection for Type Cultures (KCTC) and grown for 7 days at 37°C on HM medium (4) with 10% salts, under aerobic conditions. Genomic DNA was isolated as described elsewhere (5). In brief, cells were lysed with a mixture of lysozyme and sodium lauryl sulfate, and nucleic acids were extracted with chloroform-isoamyl alcohol (24:1 [vol/vol]), followed by DNA precipitation with ethyl alcohol. Subsequently, DNA was purified using the MEGAquick-spin Plus kit (iNtRON Biotechnology) and quantified by spectrophotometry (DeNovix DS-11 FX spectrophotometer) and fluorometry (Qubit 3.0 fluorometer). Library construction was performed using the KAPA HyperPrep kit (Roche), according to the manufacturer’s instructions. The draft genome sequence of Saccharomonospora piscinae KCTC 19743^T^ was obtained by following a complete-genome shotgun strategy (6) on an Illumina NovaSeq 6000 platform (2 × 150-bp paired-end reads) (Stab Vida, Portugal), with an output of 23,534,814 reads and a sequencing depth of 733×. Downstream analyses were carried814 out using default parameters for all software unless otherwise specified. BBDuk from the BBTools v.38.44 package (7) was employed for read quality trimming (qtrim = rl, trimq = 18) and adapter trimming (k = 21, tbo ordered cardinality). Genome assembly was performed using SPAdes v.3.13.0 (8) (option–careful). The NCBI Prokaryotic Genome Annotation Pipeline (9) was used to provide functional annotation. To determine the presence of secondary metabolite biosynthetic gene clusters, the assembled genome was analyzed using antiSMASH server v.5.0 (10).

The draft genome sequence of Saccharomonospora piscinae KCTC 19743^T^ contained 4,897,614 bp, with a G+C content of 71.0 mol%. The reported coding density was 91.82%, with 0.93 genes per kbp. The assembly resulted in 11 scaffolds (≥940 bp), with an *N*_50_ value of 1,086,926 bp and *L*_50_ value of 3. A total of 4,561 putative open reading frames (ORFs) were predicted, with an average size of 986 bp, including 4,508 coding sequences, a complete rRNA operon, 47 tRNA genes, and 3 noncoding RNA genes. The presence of the secondary metabolite biosynthetic gene clusters PKS-T1, PKS-T2, PKS-T3, and NRPS, as well as hybrid clusters, was localized in nine genomic regions within the genome sequence (Table 1). Our results suggest a high potential for Saccharomonospora piscinae to produce a variety of secondary metabolites related to the PKS and NRPS systems.

**TABLE 1:** Presence of secondary metabolite biosynthetic gene clusters in the genome sequence of *Saccharomonospora piscinae* KCTC 19743^T^, as detected using antiSMASH

Contig	Genomic region (nucleotide position, start to stop)	Biosynthetic gene cluster(s)	Most similar known cluster	Similarity (%)	MIBiG^*a*^ accession no.
1	132953 to 205507	T2-PKS	Curamycin	71	BGC0000215
	314548 to 421784	NRPS, T1-PKS	Collismycin A	7	BGC0000973
	519335 to 560387	T3-PKS	Alkyl-*O*-dihydrogeranyl-methoxyhydroquinones	57	BGC0001077
	705947 to 724544	Terpene	Isorenieratene	36	BGC0001227
	873907 to 899995	Terpene	Hopene	46	BGC0000663
2	226982 to 268169	Arylpolyene	A201A	8	BGC0001138
	286693 to 307703	Indole	Fortimicin	9	ND^*b*^
	820634 to 841380	Homoserine lactone	Albachelin	40	BGC0001211
3	1 to 9615	Ectoine	Ectoine	100	BGC0000853
	60896 to 82094	Linaridin	Lomaiviticin	6	BGC0000241
	207856 to 250926	NRPS	Sporolide	36	BGC0000150
	251706 to 297528	T1-PKS	Amycolamycin A/amycolamycin B	10	BGC0001503
	990976 to 1014377	Linaridin	ND	ND	ND
4	559645 to 802161	T1-PKS, T3-PKS	Concanamycin A	42	BGC0000040
5	47510 to 115116	β-Lactone, NRPS	Herboxidiene	8	BGC0001065
6	25520 to 47697	Terpene	Geosmin	100	BGC0000661
	168655 to 218283	Siderophore, T1-PKS	Ficellomycin	14	BGC0001593
9	1 to 13562	T1-PKS	Mediomycin A	28	BGC0001662

a MIBiG, Minimum Information about a Biosynthetic Gene cluster.

b ND, not determined.

### Data availability.

This whole-genome shotgun project has been deposited in GenBank under accession number VCEK00000000. The version described in this paper is the first version, VCEK00000000.1. The raw Illumina data from BioProject PRJNA544002 were submitted to the NCBI Sequence Read Archive (SRA) under accession number SRX7473633.
